# Exploring the emerging role of CRISPR-Cas systems in probiotic development

**DOI:** 10.1016/j.engmic.2026.100279

**Published:** 2026-06-05

**Authors:** Abrar Hussain, Naheed Mojgani, Maria Fareed Siddiqui, Mohammad Inam Khan, Syed Abid Ali

**Affiliations:** a Third World Center for Science and Technology, H.E.J Research Institute of Chemistry, International Center for Chemical and Biological Sciences (ICCBS), University of Karachi, Karachi 75270, Pakistan; b Biotechnology Department, Razi Vaccine and Serum Research Institute-Agriculture Research, Education and Extension Organization (AREEO), Karaj, Iran; c Faculty of Pharmacy, the University of Lahore, Lahore, Pakistan; d Department of Health Sciences, Saudi Electronic University, Riyadh 11673, Saudi Arabia

**Keywords:** Probiotics, CRISPR-Cas, CRISPR-based probiotics, Genome editing, Lactic acid bacteria

## Abstract

Modification of the gut microbiota by beneficial microbes can enhance an organism’s lifespan, giving rise to the concept of probiotics. Probiotics are live microorganisms that provide health benefits when taken in sufficient amounts. Owing to their outstanding health benefits, probiotics have experienced rapid expansion and gained interest for the development of new applications. The exploration of microbial applications via genetic modification is currently of great interest to researchers. Genetic engineering using the clustered regularly interspaced short palindromic repeat (CRISPR)-Cas system has received considerable attention and has established applications. Owing to these enhanced properties, the CRISPR-Cas system is currently used in medicine, agriculture, food, and biotechnology. Considering the adaptive immune system in bacteria, this genetic tool is used to alter the microbial genome. Lactic acid bacteria (LAB) are widely recognized for their probiotic potential, and over 40% of LAB species contain the CRISPR-Cas system. The rising demand for probiotics and their expanding applications necessitate the enhancement of their existing characteristics. The CRISPR-Cas system, recognized for its precision, accuracy, and speed, has enabled researchers to modify the genomes of probiotics, thereby enhancing their beneficial attributes. This system can enhance probiotic properties through additive, subtractive, or modulatory mechanisms. Various approaches have been developed to improve probiotic functionalities using the CRISPR-Cas system, such as substituting slow promoters with efficient alternatives, eliminating undesirable components, boosting metabolism, and increasing tolerance levels. Furthermore, CRISPR-engineered probiotics have emerged as next-generation probiotics with enhanced properties and advanced applications across diverse fields, including the food, medicine, agriculture, and pharmaceutical sectors.

## Graphical abstract

**Figure:**
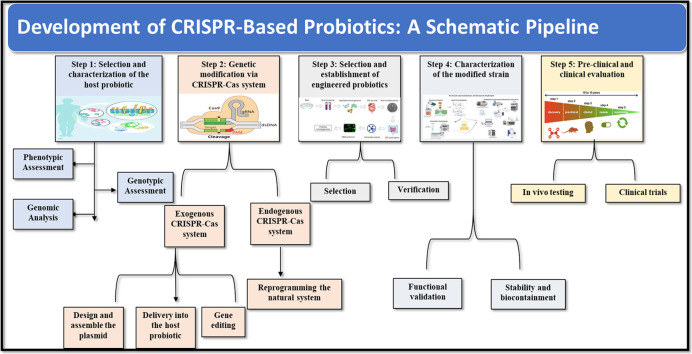

## 1 Introduction

Different genetic tools are used in various domains of the life sciences that contribute significantly to different sectors, such as disease treatment, energy production, public health, and the enhancement of microorganism functionalities. The enhancement of microbial functions and the development of desired traits via genetic tools represent areas for expanding research. Microbial genomes have been used to elucidate advanced biological functions using genetic tools. Genetic engineering tools allow the targeted modification of an organism’s genome and have diverse applications. Microorganisms with distinct metabolic flexibility and rapid growth rates are a major focus in genetic engineering [1]. A wide range of genome-editing tools has been developed, including zinc finger nucleases (ZFNs), transcription activator-like effector nucleases (TALENs), and the clustered regularly interspaced short palindromic repeats (CRISPR)-Cas systems. ZFNs and TALENs enable targeted DNA modification with varying degrees of precision, while CRISPR-Cas has emerged as a highly efficient and versatile tool for genome engineering. The transformative role of these tools in medicine, healthcare, disease detection, and biotechnology offers new methods to tackle today’s challenges and foster innovation across industries [2,3]. Among these tools, the CRISPR-Cas system is considered an advanced and precise tool with wide application in the health sector and other industries. This facilitates accurate genetic manipulation of microorganisms, enhances their functions, and paves the way for novel applications across multiple domains [3]. This system uses a single guide RNA (sgRNA) that directs the Cas9 enzyme, a protein that recognizes and cleaves DNA in a specific sequence, allowing targeted genetic modification [4].

The CRISPR-Cas system is an adaptive immune system that protects most bacteria and archaea from invading viruses, phages, and foreign genetic elements. Since its discovery in *Escherichia coli* in the late 1980s [5], it has been employed in diverse fields with significant applications [3]. Because of its simple design, high efficiency, good reproducibility, and low cost, the CRISPR-Cas system is globally recognized as a standard tool in the domain of molecular biology. Researchers are applying this tool to study human diseases, develop gene therapies, combat antibiotic resistance, engineer industrially important microbes, and improve existing microbial functionalities [6]. The health outcomes and therapeutic potential of the CRISPR system are significant, particularly when applied to the human microbiome. Because of the immense impact of microbes on host immunity, metabolism, and other physiological functions, their potential risks or benefits can intensify when specific alterations are introduced [7]. The target-specific potential of CRISPR technology within the microbiota reflects its precision and demonstrates its ability to transform human health, highlighting its therapeutic potential and health-related applications [8,9]. Additionally, this technology offers a novel approach for manipulating the human microbiome by targeting specific species and their genes, allowing the exploration of new functionalities [8]. The association of the CRISPR-Cas system with microorganisms can be classified as additive (introduction or enhancement of beneficial genes or functions), subtractive (removal or inactivation of undesirable or harmful genetic elements), or modulatory (alteration of the function of existing microbes) [10].

Probiotics have been found to move beyond traditional health benefits, paving their way into many industries, and drawing increasing attention from researchers, consumers, and the industry. This field is expanding rapidly, with a major focus on discovering and validating new strains, exploring novel applications, and advancing their use in humans and animals [11]. To date, several groups of microorganisms have been explored for their probiotic potential, dominated by lactic acid bacteria (LAB) and some fungal species such as *Saccharomyces boulardii*. The health, agriculture, biotechnology, and pharmaceutical sectors are the current targets of probiotics with proven strains and applications [12,13]. Owing to their living nature, they can be targeted for genetic manipulation to enhance their applications in the biomedical, food, feed, and agricultural sectors. In addition to conventional genetic tools, the CRISPR-Cas system is widely used and has been intrinsically identified in probiotic species [[14], [15], [16]]. The application of CRISPR-Cas systems in probiotics has opened novel and promising avenues for research, allowing precise genetic modifications that can enhance the safety, efficacy, and applications of probiotics. These modifications can also be tailored to introduce specific traits, such as the ability to prevent or treat diseases or produce valuable metabolic compounds with enhanced functions [17,18].

Investigating the function of the CRISPR-Cas system in probiotics and utilizing its potential in microbial genetics is a currently growing area of research. Besides enhancing our scientific comprehension, this emerging area has overwhelming applications in medicine, biotechnology, and pharmaceutical sciences [3,6]. Compared to conventional probiotics, genetically engineered probiotics are anticipated to have greater efficacy, improved stability, and broader functionalities [19]. These genetically altered strains have the potential for novel applications, including targeting pathogens, aiding vaccine development, enabling drug delivery, and bolstering immunity [19,20]. Ongoing research is focused on the development of probiotic strains from non-probiotic species through genetic engineering, thereby unlocking new possibilities for the development of next-generation probiotics.

With the expansion of probiotic research, it is essential to explore new applications by focusing on the utilization of genetic tools. In this regard, this review focuses on recent advancements in probiotic research using the CRISPR-Cas system. Specifically, the review describes the impact of CRISPR technology on probiotics, its role in precise genetic manipulation, and the mechanisms by which the CRISPR-Cas system enhances probiotic functionality and therapeutic potential. This comprehensive literature review highlights the synergistic interplay between CRISPR technology and probiotic science, offering promising avenues for the development of next-generation probiotics with improved efficacy and safety.

## 2 Overview of probiotics

The concept of probiotics originated when Elie Metchnikoff and colleagues observed longer life expectancies in rural populations that largely consumed fermented foods [23]. They linked this observation to the microbiota of these products and assumed that the differential microbial composition might be the reason for the longer life of those who consumed them. They proposed that changing the gut microbiota with beneficial microbes could enhance the lifespan of an individual [21]. This concept attracted experts from different fields who contributed to different aspects of probiotics, such as the identification, characterization, and functional profiling of different species. In 1965, the term “probiotic” was coined and defined as substances secreted by one microorganism that stimulate the growth of another [21,22]. Later in 2003, the term was redefined by the World Health Organization and then updated by the International Scientific Association for Probiotics and Prebiotics (ISAPP) in 2013 [23,24]. According to the ISAPP, probiotics are live microorganisms that, when administered in adequate amounts, confer a health benefit to the host [25,26]. Currently, a significant amount of literature is dedicated to describing different aspects of probiotics, such as their therapeutic potential, clinical trials, genomic analysis, and industrial applications [23,24,27].

Currently, several genera have been recognized for their probiotic potential, including *Lactobacillus, Bifidobacterium, Enterococcus, Streptococcus, E. coli, Bacillus,* and *Saccharomyces* [26]*.* Among LAB, *Lactobacillus* and *Bifidobacterium* are the most extensively studied genera, as they both hold the status of Generally Recognized as Safe (GRAS) and Qualified Presumptions of Safety (QPS). Although the genus *Enterococcus* is also recognized for its probiotic potential, it has not been granted GRAS or QPS status [[28], [29], [30]].

Probiotics exert a wide range of beneficial and health-promoting effects on the host, including the enhancement of innate and adaptive immune responses; resistance to infections; prevention and treatment of different diseases; restoration of the gut microbiota; and enhancement of the nutritional, functional, and sensory properties of food. They are also known to produce valuable metabolic compounds (termed postbiotics) such as vitamins, enzymes, and other bioactive substances, which may help alleviate the symptoms of metabolic disorders [27,31]. Additionally, emerging research highlights their role in cancer management, aging, mental health, and precision medicine [23,[32], [33], [34]].

**Fig. 1:**
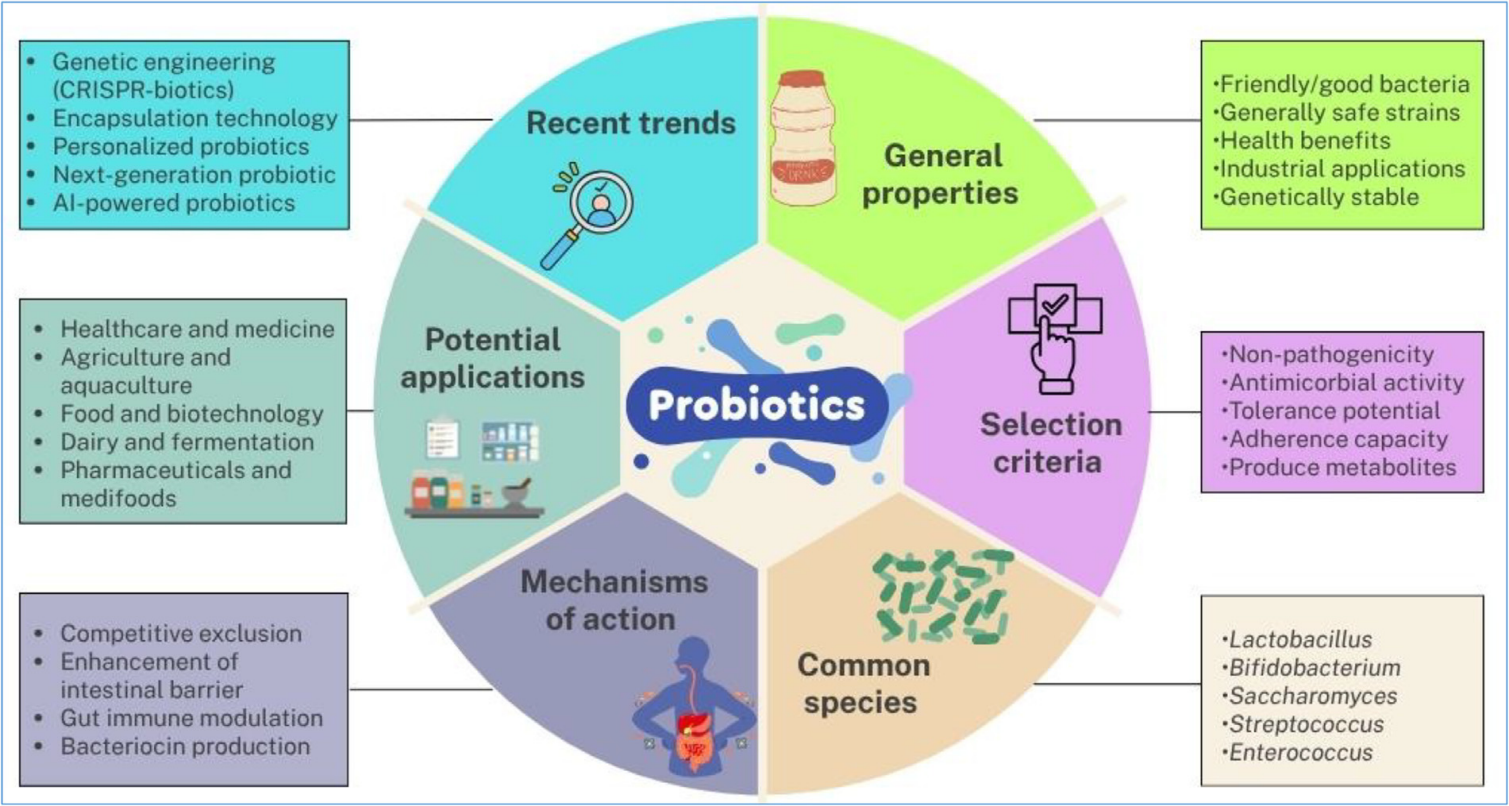
Comprehensive representation of the major dimensions of probiotics.

The documented health benefits of probiotics and their extensive use in different sectors can be attributed to proper selection. Different selection criteria have been proposed to identify microorganisms with desirable properties and the potential to serve as probiotic candidates [35]. A detailed and thorough evaluation is essential to identify a strain with probiotic characteristics. For example, selecting a probiotic strain involves the assessment of a wide range of characteristics to ensure its safety, effectiveness, and suitability for industrial applications. The important factors needed for probiotic characterization include accurate strain identification, the absence of antibiotic resistance and harmful virulence traits, and the ability to produce beneficial compounds such as postbiotics and antimicrobials. Other key factors include tolerance to pH and temperature, resistance and viability under acidic and alkaline conditions, ability to aggregate and adhere to surfaces (through hydrophobicity and attachment), and capacity to remain viable during processing [28,36,37]. Fig. 1 presents an overview of probiotics, including common microorganisms, selection criteria, mechanisms of action, health benefits, and applications in different sectors.

Although significant progress has been made in the field of probiotics, no single probiotic strain possesses all the ideal functional, safety, and technological characteristics required for its wide application. Such limitations result in a distinct disparity and show a gap between commercially available strains and those proposed or characterized in research settings [38,39]. This limits the development of new probiotic products; thus, a solution is required to fully utilize their potential. Among currently available approaches, the use of genetic engineering techniques, such as the CRISPR-Cas technology, provides promising potential. CRISPR-based engineering offers a transformative solution that enables targeted manipulation and strain-specific genetic enhancement by the addition of desirable traits, removal of unwanted characteristics, and modulation of functional pathways [14,16].

With the advent of modern genetic engineering technologies and the promising future of probiotic applications, researchers are increasingly focusing on developing innovative strategies to enhance the probiotic potential of selected strains through precise characterization, genetic improvement, and functional optimization [[40], [41], [42]]. In this context, several advanced genetic tools, such as next-generation sequencing and bioinformatics, are being applied to sequence and analyze probiotic genomes, leading to the identification of functional gaps. This targeted identification can facilitate the precise location of genetic engineering and thus help enhance probiotic attributes [43]. These approaches also assist in identifying the genetic limitations that enable the modification of specific genes, thereby enhancing their functional traits such as stress tolerance, antimicrobial activity, and host interactions [44,45]. Among these innovations, CRISPR-based probiotics have attracted significant attention. Based on the precision, efficiency, and versatility of CRISPR technology, researchers have been able to manipulate the probiotic genomes and improve their beneficial traits, and can eliminate undesirable properties, thus leading to the development of next-generation probiotics with enhanced therapeutic and functional characteristics [3,14,46,47].

## 3 Introduction to the CRISPR-Cas system

**Fig. 2:**
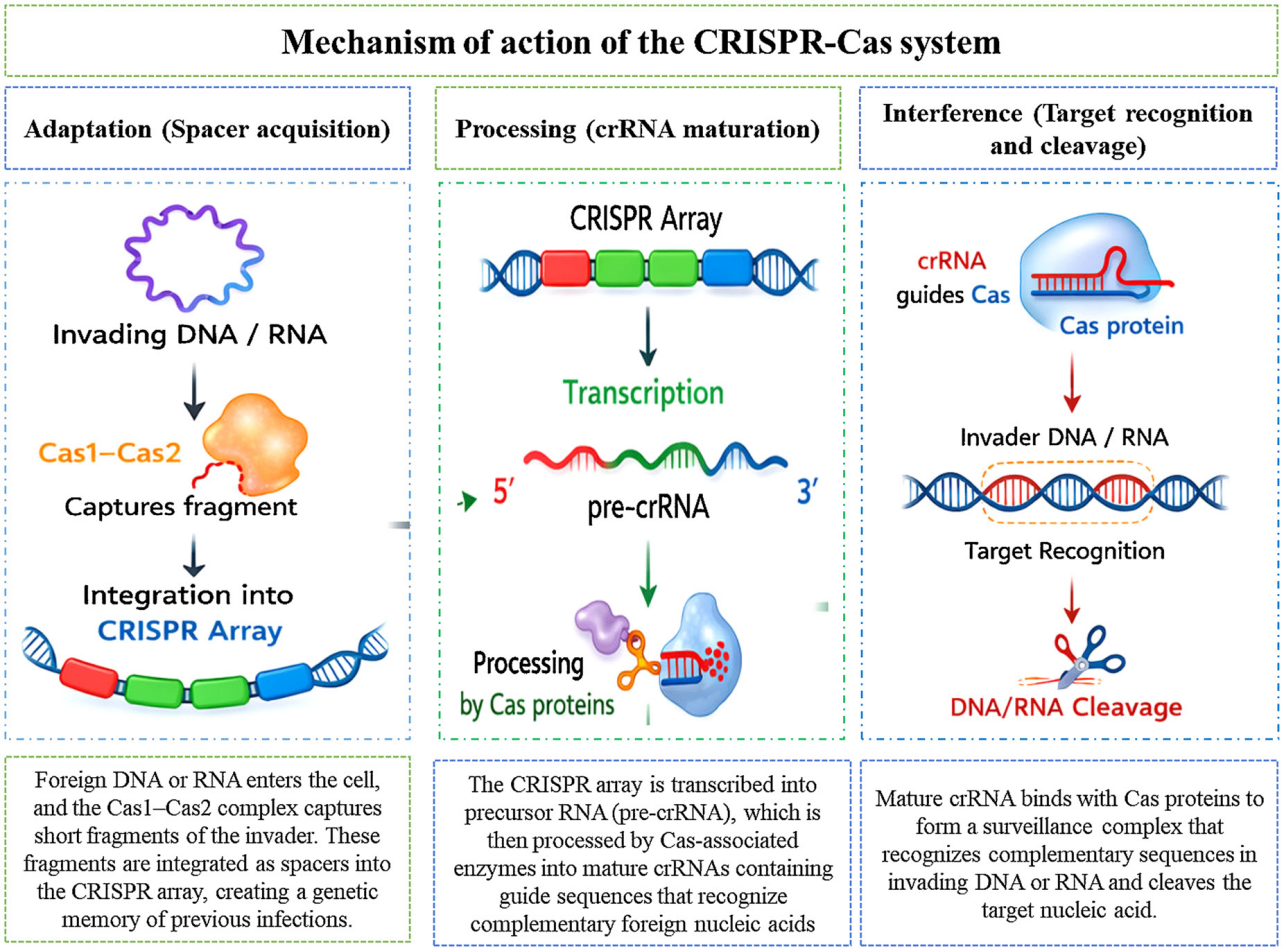
Mechanism of the CRISPR-Cas adaptive immune system.

CRISPR was first identified as short and regularly spaced repeats of approximately 30–40 base pair sequences [48]. These specific repeats form the basis of the CRISPR-Cas system, which is defined as an adaptive immune defense mechanism of bacteria and archaea that protects them from invading viruses and other mobile genetic elements [49,50]. The CRISPR-Cas system is composed of CRISPR arrays, which store genetic “memories” of past invaders, and Cas proteins, which carry out defense processes by recognizing, cleaving, or modifying DNA and RNA [6,51]. Based on their diversity, Cas proteins have recently been classified into seven types and 46 subtypes. Type I is the most prevalent in nature, and type II (including CRISPR-Cas9) is the most extensively studied because of its precision and efficiency [[52], [53],^150^]. The CRISPR-Cas system operates in three stages: (i) adaptation, during which fragments of foreign DNA (spacers) are integrated into the CRISPR array; (ii) processing, in which the array is transcribed and processed into guide RNAs (gRNAs); and (iii) interference, in which Cas proteins, guided by crRNAs, recognize and cleave the corresponding invader DNA or RNA [6]. The general mechanism of the CRISPR-Cas system is illustrated in Fig. 2. Numerous studies have explored different aspects of CRISPR-Cas systems, such as its history [5], prevalence [54], structure [55], mechanism [56], classification [57], types [58], applications [59], human usage [2], ethical considerations [60,61], and importance [48].

### 3.1 CRISPR-Cas system in LAB

In recent years, the CRISPR-Cas system has emerged as a powerful and widely adopted tool for microbial genomic engineering due to its precision, efficiency, and ability to introduce edits without leaving residual genetic markers [62]. These abilities have expanded their application to LAB, particularly to strains with probiotic features. According to the published reports, CRISPR-Cas loci are prevalent across multiple LAB genera. A previous study identified the presence of the CRISPR-Cas system in *Lactobacillus* and *Bifidobacterium* species and observed that more than half of the sequenced genomes of these genera harbored CRISPR-Cas elements, indicating that these adaptive immune systems confer significant evolutionary advantages [14,16]. This high prevalence highlights not only historical exposure to bacteriophages and mobile genetic elements, but also the importance of CRISPR-Cas in maintaining genome integrity and enabling strain-specific defense mechanisms [15,63]. The diversity of CRISPR-Cas systems in LAB species provides insights into their niche-specific adaptations and evolutionary paths in environments such as the gastrointestinal tract and fermented foods [64]. Type II systems are the most common in LAB species, with subtype II-A being markedly widespread [14,69]. The functional relevance of CRISPR-Cas extends beyond phage resistance; in some cases, its presence is correlated with enhanced safety and probiotic potential. Several studies have shown that in *Enterococcus* spp., an inverse association exists between CRISPR-Cas loci and antibiotic resistance genes, suggesting that CRISPR-Cas may restrict the acquisition of resistance determinants via horizontal gene transfer [65,66,151].

In *Enterococcus faecalis*, distinct loci such as CRISPR1-Cas and CRISPR2 were first characterized by Palmer and Gilmore [67], whereas *Enterococcus faecium* isolates from spring water possess compact CRISPR arrays of approximately 190 bp with conserved repeat regions 24 bp long [68]. In a study by Hussain et al, multiple CRISPR-Cas-associated genes—CRISPR-Cas1, CRISPR3, CRISPR-Cas3/csn1, and CRISPR2—were identified via polymerase chain reaction (PCR) analysis in enterococcal strains with probiotic attributes [30]. In a similar study, Crawley et al analyzed 1262 publicly available *Lactobacillus* genomes and observed the widespread prevalence of subtype II-A [69]. The implementation of CRISPR-Cas tools for *Lactobacillus* species is now well-established, supporting both functional genetics and strain improvement [20]. In L. *lactis*, a single-plasmid–inducible CRISPR-Cas9 system (pNZCRISPR) was developed using two nisin promoters, the first to drive Cas9 expression and the second to drive sgRNA transcription [164]. Similarly, when using the plasmid pLCNICK with Cas9^D10A^ (nickase) in the genome editing of L. *casei*, reduced lethality and enhanced efficacy (62%) were observed [75,165]. A deeper understanding of the diversity of CRISPR-Cas systems in LAB could significantly contribute to their practical application, especially in food production, where they can assist in controlling microbial communities, increasing phage resistance, and enhancing strain robustness. This system could contribute to the development of next-generation probiotics [66]. However, despite these advantages, the precision of the CRISPR-Cas system is not absolute. Off-target effects and variability across microbial species can compromise reproducibility and safety. In applied settings such as probiotics, regulatory concerns and the potential ecological impact of engineered strains warrant careful consideration, limiting the straightforward translation of CRISPR-based modifications to clinical or commercial use.

## 4 Development of CRISPR-based probiotics

Recent breakthroughs in gene-editing technologies have greatly expanded the applications of CRISPR-Cas systems, moving beyond their natural role in protective immunity to therapeutic and industrial uses. In microbial biotechnology, CRISPR technology is now widely applied to enhance microbial traits such as stress tolerance, metabolic efficiency, and overall performance [3,17]. Over the past few decades, genome editing has been performed on *Lactobacillus* and *Bifidobacterium* species. Earlier approaches, such as plasmid-mediated homologous recombination or prophage recombinase-assisted editing using double- or single-stranded DNA (dsDNA and ssDNA, respectively), were limited by issues such as unstable mutations, narrow host ranges, temperature sensitivity, and inefficient editing [70]. With the emergence of the CRISPR-Cas system, this genome-editing tool has been widely employed by researchers, especially for probiotic bacteria, such as *Lactobacillus* and *Bifidobacterium* species [70]. CRISPR-based strategies have also been reported to improve the probiotic properties of a strain by increasing its resilience to biotic and abiotic stressors, such as bile salts, acidic and oxidative environments [16]. Moreover, the presence of CRISPR-Cas loci in probiotic strains is increasingly recognized as a critical genomic feature because of its contribution to genome stability, adaptation, and enhanced survival under hostile conditions, supporting its probiotic potential [16,71]. However, the presence of CRISPR-Cas loci does not necessarily correlate with functional immunity. Many loci are inactive, incomplete, or poorly expressed under normal physiological conditions. Although CRISPR systems can limit horizontal gene transfer, they may also restrict the acquisition of beneficial traits, potentially constraining probiotic adaptability and development. Table 1 summarizes the presence of CRISPR-Cas systems in different probiotic strains and emphasizes the key enhancements in their probiotic properties, along with their anticipated results.

**Table 1:** Summary of CRISPR-Cas systems in probiotic strains, exploring the types, targeted probiotic properties, and potential applications.

Probiotic Strain	Detected CRISPR-Cas Type(s)	Used CRISPR-Cas System	Enhanced Probiotic Properties	Potential Applications	References
*Lacticaseibacillus rhamnosus* GG	Type II-A	CRISPR-Cas9	Increased bile salt and acid resistance and better epithelial adhesion	Functional foods, gut health, and IBD therapy	[72,73]
*Lacticaseibacillus casei* BL23	Type II-A	CRISPR-Cas9D10A nickase system (exogenous)	Optimized lactic acid production and resistance to pH	Fermented, dairy, and synbiotic foods	[[74], [75], [76]]
*Lactiplantibacillus* *plantarum* WCFS1	Type II-A	CRISPR-Cas9	Deletion of some genes improved the oxidative stress tolerance	Food preservatives and mucosal vaccine delivery	[20,77]
*L. acidophilus* NCFM	Type I	CRISPR loci	Phage resistance and genetic stability	Gut probiotics and dairy products	[[78], [79], [80]]
*Lactobacillus delbrueckii* subsp. *bulgaricus* ATCC 11,842	Type II-A	CRISPR2	Enhanced fermentation robustness and high exopolysaccharide production	Yogurt and probiotic beverage fermentation	[[81], [82], [83]]
*Lactobacillus brevis* ATCC 367	Type I	RecE/T assisted CRISPR Cas9	Phage immunity, improved performance in fermentation	Kombucha, fermented vegetables	[84,85]
*Bifidobacterium longum* NCC2705	Type I-C	CRISPR-Cas9	Acid and bile salt tolerance and mucosal adhesion	Infant gut colonization, prebiotic formulation	[[86], [87], [88]]
*Bifidobacterium breve* UCC2003	Type I-C	CRISPR array (Cas4, Cas1, and Cas2)	Enhanced carbohydrate metabolism and adhesion to intestinal epithelium	Pediatric probiotics	[89,90]
*Bifidobacterium animalis* subsp. *lactis* BB-12	Type I-E	Bala1 CRISPR locus	Potentially modify the immune responses	Synbiotics and dairy supplements	[[91], [92], [93]]
*Streptococcus thermophilus* LMD-9	Type II	CRISPR3, CRISPR1, and CRISPR2	Phage immunity and robust fermentation traits	Yogurt and cheese production	[[94], [95], [96]]
*Limosilactobacillus reuteri*	Type II-A	CRISPR-Cas9	Enhance the immune regulation, antimicrobial activity, and adhesion to the gut lining	Food fermentation	[10,20,97]
*Lacticaseibacillus casei*	Type I-E, I-C	CRISPR-Cas9D10A	Enhanced acid and bile resistance and produce metabolites	Lactic acid production and designer probiotics	[74,75]
*Enterococcus faecalis* OG1RF	Type II	CRISPR loci (CRISPR1-cas and CRISPR2)	Control over plasmid transfer, reduced ARG dissemination	Probiotic safety in clinical formulations	[[98], [99], [100]]
*E. coli* Nissle 1917	Type I-E	CRISPR-Cas9 (engineered)	Engineered as a therapeutic delivery system targeting pathogens in the gut	Synthetic biology, antimicrobial delivery, and IBD therapy	[[101], [102], [103]]

One of the most significant advantages of CRISPR-Cas technology is its ability to engineer probiotic strains with enhanced resilience to gastrointestinal stressors, including low and high pH, bile salts, and antimicrobial compounds that are naturally present in the gut. These targeted modifications improve the survival and colonization efficiency of engineered probiotics, enabling more effective persistence within the host. Beyond stress tolerance, CRISPR tools also make it possible to adjust traits that influence how probiotics behave in the intestinal environment, such as their ability to attach to host tissues, use available nutrients, and interact with the immune system [[104], [105], [106]]. These types of editing allow engineered strains to compete more successfully with pathogens and persist in the gut for longer periods.

**Fig. 3:**
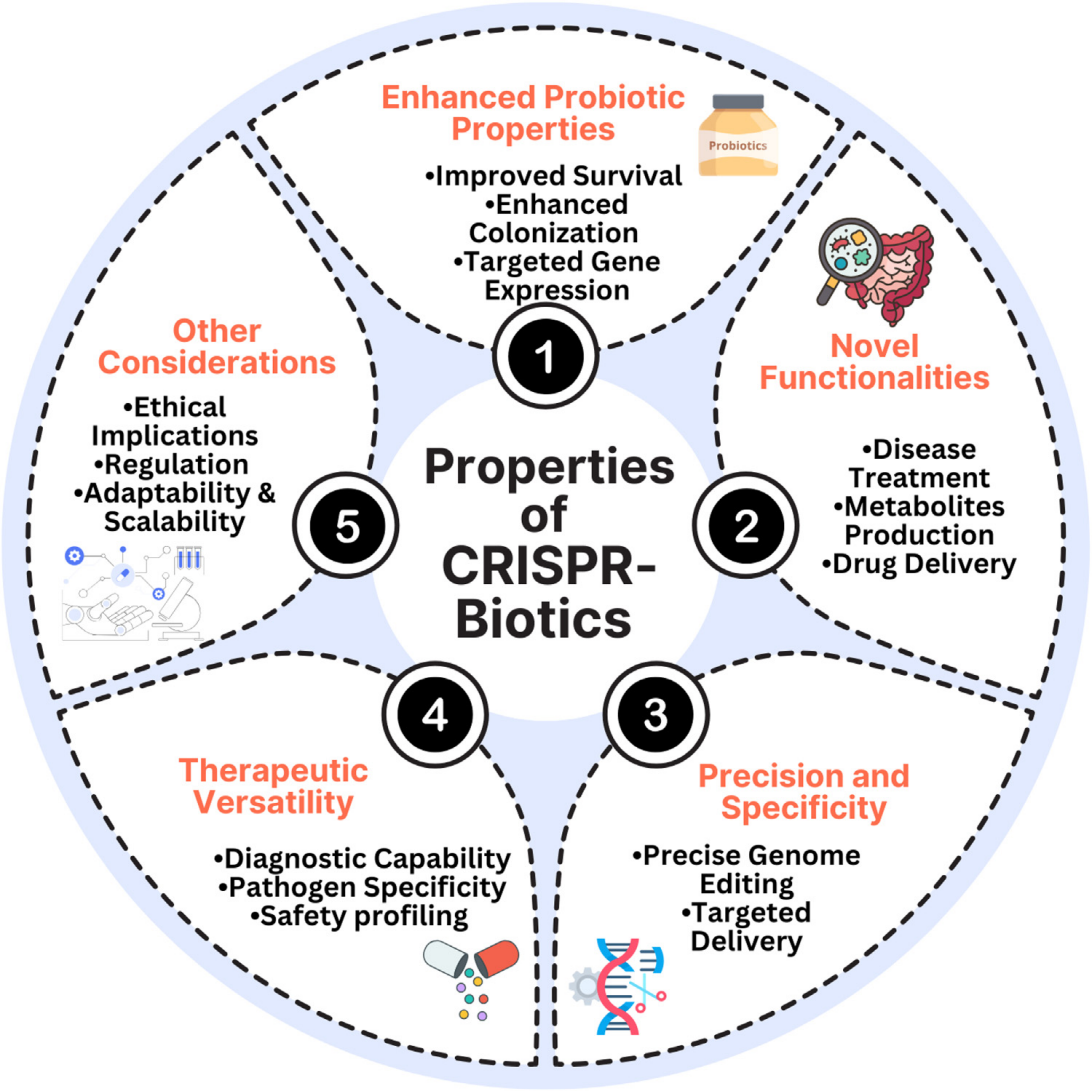
General properties and functional attributes of CRISPR-biotics.

The diverse interactions between probiotics and the CRISPR system have led to the emergence of CRISPR-based probiotics, a term that signifies the application of the CRISPR-Cas system and its impact on probiotic strains [6]. Interestingly, bacterial species with recognized probiotic potential often carry more diverse CRISPR-Cas systems than those not associated with probiotic potential [107]. However, this correlation should be interpreted with caution, because the number of CRISPR-Cas loci does not necessarily reflect their functionality and efficacy. In recent years, research has increasingly focused on the development of CRISPR-based probiotics with advanced properties compared to traditional probiotics [108]. According to previous studies, a greater number of CRISPR-Cas loci may be correlated with beneficial traits. For example, enterococcal strains with more CRISPR elements are less prone to acquiring phages and mobile genetic elements [109]. Fig. 3 summarizes the main features and advantages of CRISPR-based probiotics.

**Fig. 4:**
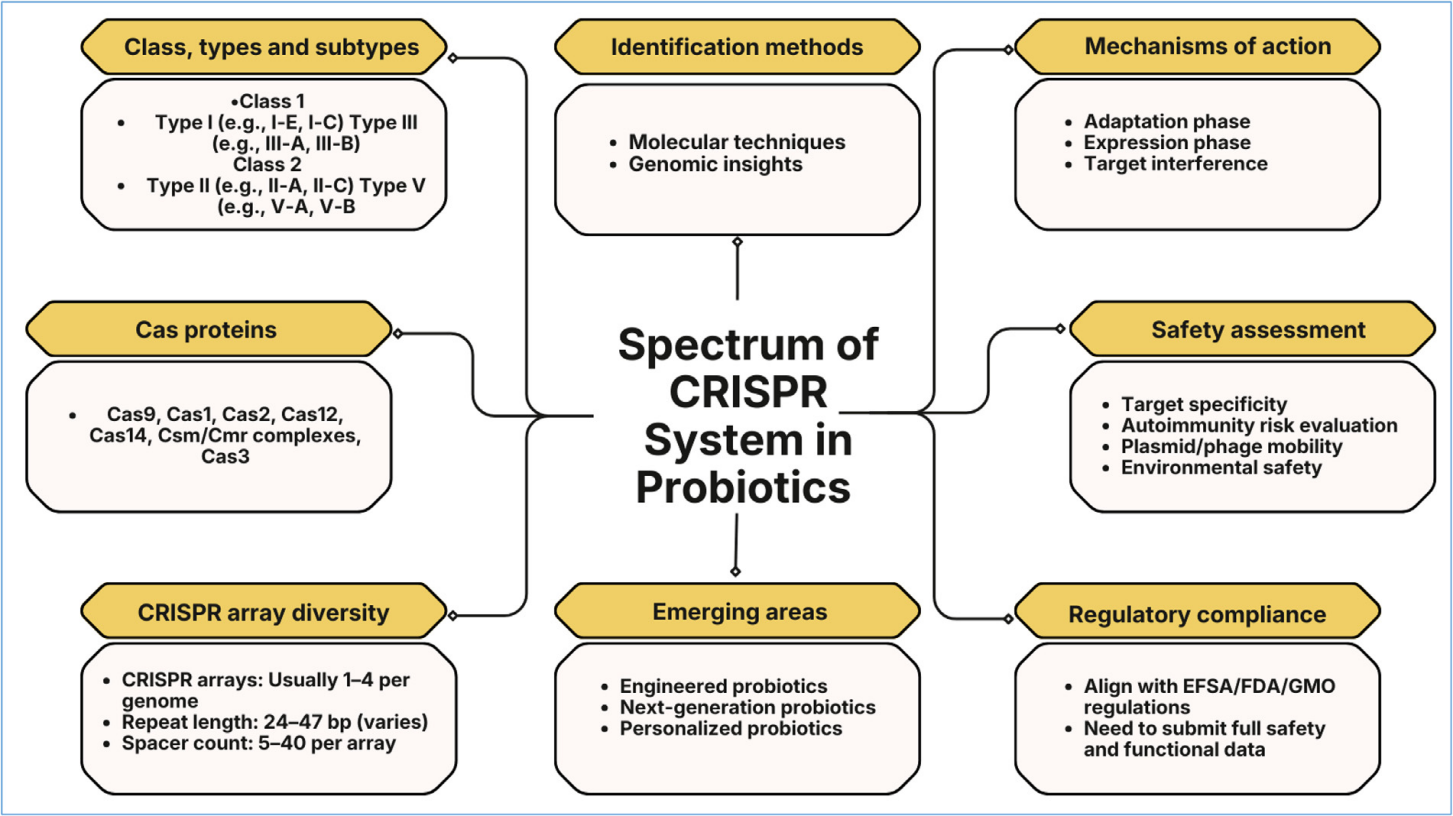
Overview of the CRISPR-Cas systems in probiotics, highlighting types, Cas genes, mechanisms, identification, and emerging areas.

The genome of the well-known probiotic strain *Lactobacillus rhamnosus* Pen has a CRISPR locus of 1092 nucleotide regions and 16 spacers that are 30 nucleotides long. The locus belongs to class II and is composed of the *cas1, cas2, cas9*, and *csn2* genes, and one CRISPR array. The presence of these genes was found to have a role in resistance against bacteriophages [110,111]. Previously, successful attempts were made to edit the probiotic *Clostridium butyricum* genome using the type II CRISPR‐Cas9 system, although challenges, such as the toxicity of the Cas9 nuclease and the low transformation potential of the strain, were present [112,113]. These limitations can be overcome by using various approaches. Recently, Kumari et al. (2026) presented an *in silico* framework for the rational enhancement of probiotic strains through a CRISPR-Cas9–guided design, while integrating structural bioinformatics and immunoinformatic analyses. Their study provided a computational foundation for designing safer and more effective probiotic strains for gut health, immunomodulation, and disease prevention [162]. Similarly, CRISPR-Cas9–based editing has been demonstrated in L. *reuteri* in conjunction with ssDNA recombination, whereas dsDNA recombination was used in *Lactiplantibacillus plantarum* [167,168]. The different spectra of the CRISPR-Cas system in probiotics are summarized in Fig. 4.

### 4.1 Practical implications of CRISPR-Cas systems in probiotics

In probiotic research, CRISPR-Cas tools are generally applied in three main ways: introducing new functions (additive), removing unwanted traits (subtractive), or adjusting existing pathways (modulating). Using these strategies, genes that confer benefits can be inserted, harmful or undesirable genes can be deleted, and the expression of target genes can be upregulated or downregulated to achieve the desired function [71]. For instance, the CRISPR tool is used to eliminate the lipoteichoic acid synthase (*lta)* gene in probiotic *L. acidophilus* by using an exogenous nickase called nCas9^D10A^, which leads to a change in its immunomodulatory profile [20,75,114]. Tolerance to stress and sugar catabolism by probiotic strains is also enhanced by editing their genes using CRISPR tools to optimize fermentation conditions [14]. Similarly, bile salt hydrolase potential can be improved using the CRISPR-Cas system. In one study, the native promoter of the *bshA* gene was substituted with the stronger pgm promoter, which led to higher transcript levels and increased enzyme activity [52]. Such modifications can improve gut persistence and metabolic performance. Adjustments in genes involved in carbohydrate metabolism have also been linked to improved adhesion and colonization in the intestine [107]. For instance, the development and optimization of CRISPR-Cas9 coupled with ssDNA was experimentally validated in the *Lactobacillus reuteri* ATCC PTA 6475 strain. In this study, the authors documented 90–100% recovery efficiency after targeted mutations, deletions, and codon modifications [163].

The adaptability of the bacterial genome, combined with the precision of the CRISPR-Cas system, offers excellent opportunities for probiotic strains to be used as starter cultures in fermentation or functional foods [52]. For instance, the overexpression of chaperones such as *gro*ESL in *Lacticaseibacillus paracasei* and *Bifidobacterium breve* was found to enhance species survivability under high temperatures, stress, and the harsh gut environment [115]. Recently, Raftopoulou et al. (2026) reported that >95% of the available *B. lactis* genomes harbor a conserved Type I-G CRISPR-Cas system, which can be used to develop and validate a broadly applicable genome-editing framework [170].

Similarly, the CRISPR-Cas system has been used to enhance the expression of genes linked to probiotic efficacy, including the overexpression of mucus-binding proteins, production of exopolysaccharides, and genes that promote gut colonization. In addition, the CRISPR-Cas system, via gene knock-in mechanisms, enables the introduction of sugar catabolism genes into probiotic strains, broadening the metabolic capabilities of engineered strains [115]. These applications illustrate how CRISPR-Cas tools can be used to create more robust, competitive, and functionally targeted probiotic candidates. However, the translation of CRISPR-enabled modifications into industrial and food-grade applications faces several constraints. Regulatory frameworks in many regions impose strict limitations on genetically modified microorganisms, particularly in food systems. Additionally, the engineered traits may not remain stable under large-scale fermentation conditions or during long-term storage. There is also a risk that laboratory-optimized modifications may not perform consistently in complex real-world microbial ecosystems, potentially affecting product quality or safety. Therefore, robust validation under industrial conditions, careful consideration of regulatory factors, and consumer acceptance are essential.

### 4.2 Experimental identification of CRISPR-Cas system and genes in probiotic strains

To comprehensively investigate the spectrum of CRISPR-Cas systems in probiotic strains, it is essential to integrate both experimental and computational approaches and use this synergistic approach to apply this system to the desired organisms. Molecular techniques, including PCR, can amplify the target genes of the CRISPR-Cas system. PCR analysis provides a strong basis for the identification, characterization, and functional analysis of CRISPR-Cas systems in various probiotic species. Genomic approaches, such as whole-genome sequence analysis with bioinformatics tools, such as CRISPRFinder and CRISPRCasFinder, can also facilitate the *in silico* detection and annotation of CRISPR loci and associated Cas genes in probiotics.

#### 4.2.1 Molecular identification of the CRISPR-Cas system in probiotics

PCR is a widely used molecular technique for identifying CRISPR-Cas genes in probiotic strains [116]. Using specific Cas gene primers, PCR amplification can target conserved regions within the DNA of probiotic species, which are then used for the identification and confirmation of specific Cas genes, such as *cas1, cas2, cas5*, and *cas9*. PCR amplification offers a rapid and sensitive method for screening for the presence of CRISPR-Cas genes in both traditional and novel probiotic strains. A new technique called CRISPR-typing PCR (ctPCR), developed by Wang et al. (2018), has also been used for the detection and typing of DNA based on the Cas9 nuclease [117]. Studies have demonstrated the experiential confirmation of CRISPR-Cas genes using a PCR-based approach [118]. CRISPR-Cas genes were also identified in lead *E. faecium* probiotic strains by Hussain et al, who observed a greater prevalence of CRISPR3 and CRISPR-Cas3 csn1 while a lower prevalence of CRISPR2 in the chosen strains [30].

#### 4.2.2 Genomic insight on CRISPR-Cas genes, spacer sequences, and arrays in probiotic strains

Molecular assessment of the CRISPR-Cas system via PCR, although widely used and highly specific, poses several challenges, including cost, time, and artifact errors. In contrast to PCR analysis, the genomic analysis using publicly available tools and databases offers a reliable, rapid, inexpensive, and easily accessible method for identifying CRISPR-Cas systems in bacterial genomes, including probiotic strains [119]. The genomic analysis of probiotic strains, commonly called probiogenomics, is currently used in various applications, with results being obtained faster, and more data is analyzed in less time [120]. Various online tools have been used to detect and identify CRISPR/Cas genes in microbial genomes. For instance, Rostampour et al. (2024) analyzed 675 publicly available sequences of L. *plantarum* in terms of the diversity, occurrence, and evolution of the CRISPR-Cas system. This study investigated the presence, structural variations, phylogenetic relationships, and diversity of CRISPR-Cas systems and revealed that 143 strains harbored confirmed CRISPR-Cas systems, with subtype II-A being predominant [169]. A summary of the online tools and databases used for the identification, detection, and prediction of the CRISPR-Cas system in bacterial genomes, including probiotic strains, is provided in Table 2. In addition to these tools, other sources, such as Proksee, have the option of a CRISPR-Cas finder [121]. These tools facilitate the identification of CRISPR-Cas genes, arrays, and spacer sequences in bacterial genomes.

**Table 2:** Summary of various databases and online servers that help to identify and explore the presence of the CRISPR-Cas system and genes in microorganisms.

Tools, databases, or webservers	Description	Link	References
CRISPRCasFinder [online]	The CRISPRCasFinder program enables the easy detection of CRISPRs and Cas genes in user-submitted sequence data. This is an update of the CRISPRFinder program with improved specificity and indication of the CRISPR orientation.	https://crisprcas.i2bc.parissaclay.fr/CrisprCasFinder/Index	[122]
CRISPRCasMeta [online]	CRISPRCasMeta is a workflow dedicated to the research and annotation of CRISPR/Cas structures in metagenomic data. It uses CRT and MetaFinder to perform analysis.	https://crisprcas.i2bc.paris-saclay.fr/CrisprCasMeta/Help#crispr	[122].
CRISPRCasdb	The CRISPRCasdb provides access to both CRISPRs and Cas genes. This tool uses CRISPRCasFinder and determines the system’s type and subtype to process public whole genome assemblies.	https://crisprcas.i2bc.paris-saclay.fr/MainDb/StrainList	[123]
CRISPRFinder	A web tool that identifies CRISPRs, including the shortest ones (one or two motifs), defines DRs and extracts spacers, gets the flanking sequences to determine the leader, and blasts spacers against the GenBank database.	http://crispr.u-psud.fr/Server/CRISPRfinder.php	[124]
MacSyFinder	A program that is used to mine genomes for molecular systems with an application to the CRISPR-Cas system	https://github.com/gem‑pasteur/macsyfinder	[125]
CRISPRCasTyper	An automated tool for the identification, annotation, and classification of CRISPR-Cas loci	https://crisprcastyper.crispr.dk/#/submit	[126]
CRISPRidentify	CRISPRidentify is a tool that identifies the CRISPR arrays using a machine learning approach.	https://github.com/BackofenLab/CRISPRidentify	[127]
CRISPRimmunity	CRISPRimmunity (CRISPR-associated Important Molecular events and Modulators Used in geNome edITing identifY), a CRISPR-oriented web tool to dissect the key molecular events during the co-evolution of CRISPR and anti-CRISPR mechanisms.	http://www.microbiome-bigdata.com/CRISPRimmunity/index/	[128]
CRISPR comparison toolkit (CCTK)	A command-line software toolkit that is used for the rapid identification, visualization, and analysis of CRISPR array diversity.	https://crispr-comparison-toolkit.readthedocs.io/en/latest/	[129]
PILER-CR	An online tool used for the fast and accurate identification of CRISPR repeats in bacterial genomes.	https://www.drive5.com/piler/	[130]

## 5 Importance and applications of CRISPR-based probiotics

**Fig. 5:**
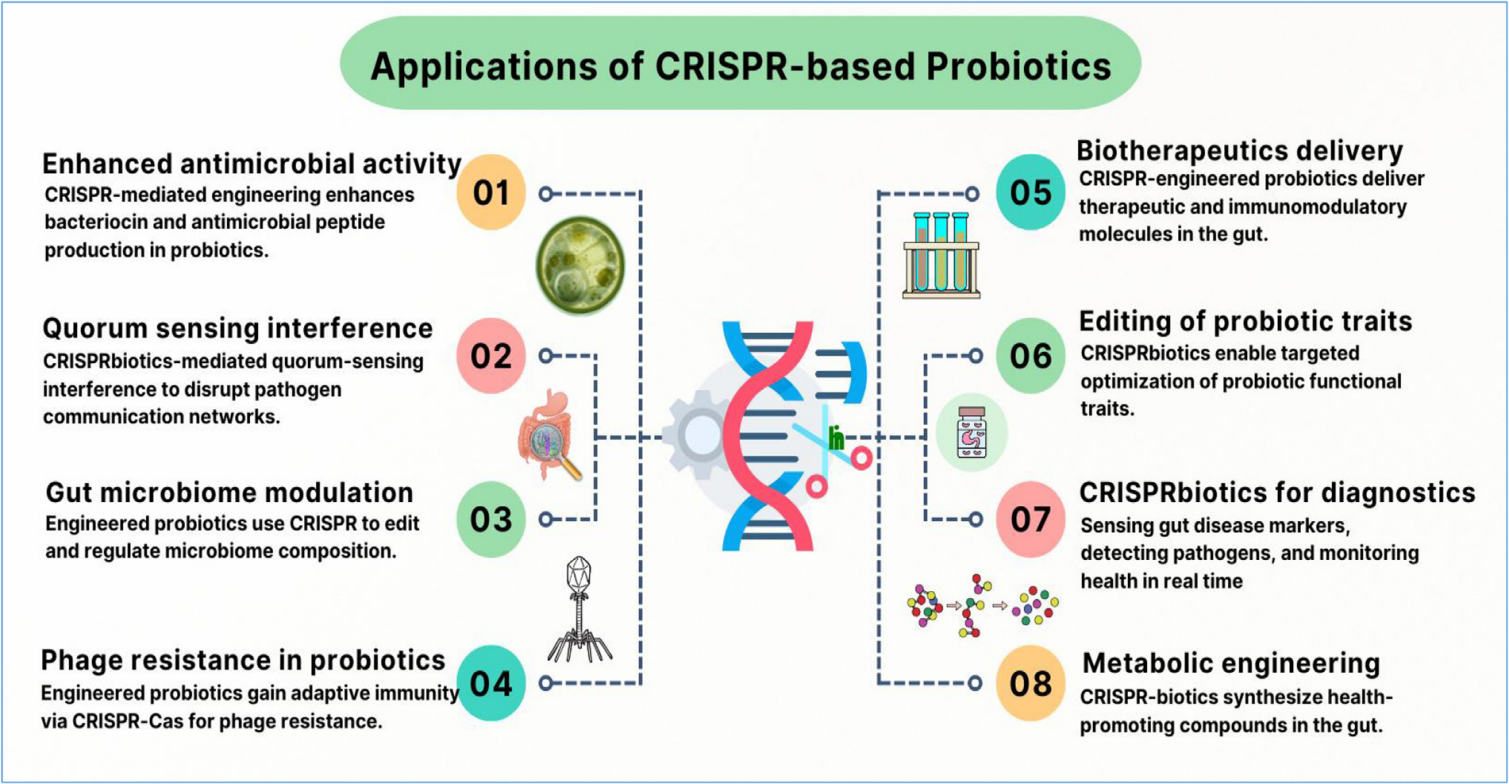
Potential applications of CRISPR-engineered probiotics in various sectors.

The concept of CRISPR-based probiotics is growing rapidly and has advanced properties, making them an important agent across various sectors with different functions and applications. In academic research, CRISPR-engineered bacteria have been used to detect specific biomarkers, particularly for disease diagnosis. Researchers are currently trying to design or develop a probiotic strain using CRISPR technology that has desirable properties, such as greater survivability, enhanced tolerance, and good immunomodulatory potential [108]. The development of CRISPR-based probiotics with greater therapeutic potential is also under investigation. CRISPR technology is also used to remove or inactivate genes that play a role in bacterial virulence and pathogenicity, thereby enabling the development of safer and more active probiotic strains [108]. Zhong et al. (2023) used the probiotic strain L. *rhamnosus* GG (LGG) and identified a type II Cas9 called LrCas9, which was successfully applied as a plant genome-editing tool. LrCas9 has efficient genome editing capacity in various plants, such as rice, wheat, and tomato, with high fidelity [72]. The yeast strain *Saccharomyces cerevisiae* BS016, which expresses the human P2Y2 purinergic receptor, senses pro-inflammatory molecules via the CRISPR-Cas9 system [16]. Similarly, a *Bacillus subtilis* strain (BsS-RS06551) edited using CRISPR-Cas9 exerted a positive effect on obesity and metabolism [16]. Recently, Xie et al. (2026) presented an endogenous type II-A CRISPR-Cas genome editing workflow for LGG designed for functional strain construction and generated a β-glucuronidase (GUS)-expressing LGG strain [73]. CRISPR technology in precision microbiome engineering offers a safe way to eliminate genes encoding pathogenic traits and improve the beneficial properties of various microbes [131]. Additionally, CRISPR offers a host-specific and precise editing system that reduces off-target effects and enhances probiotic efficiency [132]. Fig. 5 summarizes the major applications of CRISPR-based probiotics and highlights their potential roles in various sectors.

### 5.1 Therapeutic applications

The therapeutic applications of probiotics are well-known. Several studies have demonstrated the therapeutic, disease-managing, and treatment roles of probiotics [23,[133], [134], [135]]. However, the therapeutic potential of CRISPR-based engineered probiotics remains largely underexplored, and there is increasing interest in their application for the treatment and prevention of different diseases [16]. For instance, the butyrate-producing probiotic strain *B. subtilis* BsS-RS06551, engineered using CRISPR technology, was used in an animal model of obesity and exhibited promising results [136]. Similarly, CRISPR-based genetically engineered probiotics have been used for vaccinations and were shown to enhance immune reactions [137,152,153]. The engineered *Propionibacterium freudenreichii* strain, which releases vitamin B12 and short-chain fatty acids (SCFAs), was found to reduce hepatic steatosis in mouse models of non-alcoholic fatty liver disease [153]. Although engineered probiotics have potential applications in medical settings, careful consideration should be given to their long-term efficacy and potential impact on their environmental release [166].

### 5.2 Delivery applications

Probiotics are widely used as vehicles for delivering various therapeutic agents to the gut. However, certain challenges limit their delivery applications; thus, a protective mechanism must be provided for their safe delivery [138]. Studies have revealed the importance of the CRISPR-Cas system in probiotic engineering. For instance, Neil et al. (2021) developed a high-efficiency conjugative delivery vehicle for CRISPR-Cas9 (conjugative probiotic) that was used to eliminate approximately all antibiotic-resistant *E. coli* strains from the gut of a mouse model using a single dose [104]. However, challenges such as maintaining cell viability during processing and storage and achieving targeted colonization and functional activity at the desired site still exist. Additionally, there is a limited understanding of how CRISPR-modified strains interact with native microbiota and host immune responses after delivery. Therefore, integrating advanced delivery technologies with CRISPR-based engineering is essential to fully realize the therapeutic and functional potential of next-generation probiotics.

### 5.3 Enhanced metabolite production

Probiotics produce different metabolites (postbiotics), including SCFAs. These metabolites play a crucial role in the gut-brain axis and neurological disorders [23]. The use of CRISPR-Cas systems allows the editing of probiotic bacteria that can produce more SCFAs (by modifying specific metabolic pathways) such as propionate and butyrate, thereby contributing to a healthy gut microenvironment [131]. However, there is a gap in the complexity of metabolic networks in probiotic bacteria, where single-gene edits may not translate into predictable phenotypic effects owing to pathway interconnectivity and regulatory feedback mechanisms. There is also a limited integration of systems biology approaches (*e.g.*, multi-omics and metabolic flux analysis), which are essential for optimizing metabolite production. Therefore, future studies should focus on combining CRISPR-based editing with a system-level design to elucidate its full potential for enhancing probiotic functionality.

### 5.4 Enhanced metabolic activities

The use of the CRISPR technology in probiotic strains offers a promising method for modifying certain genes involved in metabolism. The precise editing of glucose-regulating enzymes offers a way to better regulate glucose levels. Studies have shown that CRISPR-edited systems enhance metabolic activity in probiotics [131,139]. However, such effects depend on several interconnected pathways rather than on a single-gene editing pathway.

### 5.5 Next-generation probiotics

The use of the CRISPR-Cas system is highly recommended for designing next-generation probiotics [45,52,154]. These next-generation probiotics are used to boost animal growth, optimize feed conversion, and competitively exclude pathogens [40]. Recently, commercial probiotics have been found to have more CRISPR genes, whereas strains of clinical origin have been found to have fewer or no CRISPR genes, indicating an inverse relationship between CRISPR and antibiotic resistance genes [141]. This area is growing rapidly but faces several challenges in safety assessments, host interactions, and *in vivo* validation studies.

## 6 Limitations and challenges of using the CRISPR-Cas system in probiotics

Although the CRISPR-based engineering of probiotics has opened up new possibilities, its practical use is still restricted by technical, biosafety, and regulatory constraints. One major hurdle is the efficient delivery of CRISPR-Cas components into the host strain. Transformation efficiency varies widely and depends on factors such as cell wall structure, growth phase, strain density, plasmid backbone, and the parameters used during electroporation or chemical transformation (such as pulse strength, buffer type, or incubation time) [16,140,153]. These differences often require species-specific protocols, and transformation remains extremely difficult in some genera. Some limitations of this technology are listed below.

The use of antibiotic resistance markers in microbial engineering adds another layer of complexity. Selecting markers that are incompatible with the host conditions can lead to poor recovery of edited strains or rapid elimination *in vivo*. Safety concerns are also raised when antibiotic resistance genes introduce the possibility of horizontal transfer to commensals or pathogens [8,52]. Contamination during the editing process can further compromise the strain identity, particularly when handling non-model probiotic species.

Biosafety is another fundamental issue because CRISPR-edited strains do not have a long history of safe use. Different types of built-in contamination associated with kill switches, metabolic auxotrophies, and nutrient dependencies are currently encountered by CRISPR-edited strains [70].

Clinical validation remains limited, as the currently used CRISPR-edited probiotics are only validated using *in vitro* or *in vivo* systems or preclinical *in vivo* models. Additionally, the absence of a standardized protocol for the evaluation of the colonization ability, genetic stability, immunogenicity, or unintended effects of engineered probiotic strains in the gut environment limits the number of clinical trials. Thus, extensive preclinical safety testing and phased clinical trials are needed before clinical adoption is feasible [16,70].

The unavailability of established regulatory frameworks poses a challenge for the development of CRISPR-engineered probiotics. The approval process for genetically modified organisms (GMOs) differs among countries and often lacks specific guidelines, particularly for CRISPR-based probiotics. Review bodies must consider not only edited organisms, but also the editing methods, containment strategies, delivery systems, and ecological impacts [70,115]. Until regulatory expectations become clearer and harmonized, translation into the market or clinic will remain slow.

The translation of CRISPR-based probiotics from laboratory validation to therapeutic implementation is currently hampered by a limited number of *in vivo* studies, which often rely on small sample sizes. This limitation reflects the nascent stage of this technology and presents significant challenges for assessing its safety, efficacy, and long-term consequences in complex living systems [158].

**Fig. 6:**
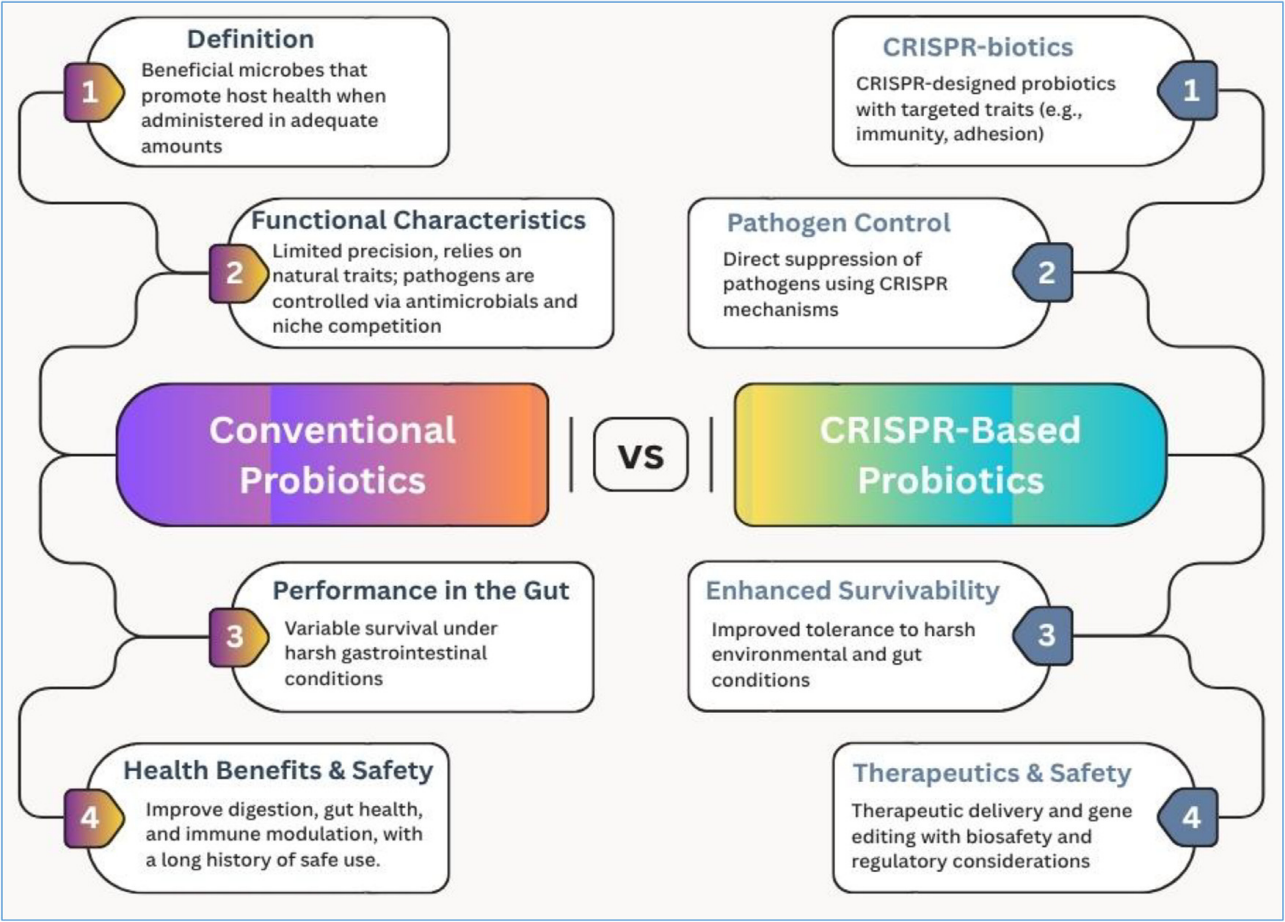
Comparative overview of conventional probiotics and CRISPR-engineered probiotics (CRISPR-biotics).

A comparison between traditional and CRISPR-based probiotics is shown in Fig. 6. Conventional probiotics provide natural health benefits, such as gut colonization, digestion support, and immune modulation, but their functions remain strain-dependent and imprecise. In contrast, CRISPR-based probiotics allow for targeted modification, pathogen-specific inhibition, enhanced survivability, therapeutic versatility, dynamic microbiome modulation, optimized metabolite production, and integrated biosensing functions. However, while conventional probiotics are generally recognized as safe and widely accepted with a known usage history, CRISPR-biotics require biosafety standards and regulatory approval before their large-scale clinical and commercial use can be allowed.

## 7 Advancement of CRISPR-based probiotics over other engineered probiotics

CRISPR-edited probiotics offer significant advancements over other engineered probiotics (edited with other genetic tools), mainly because of the efficient, accurate, and precise nature of the CRISPR-Cas system. CRISPR-Cas9/Cas12a systems allow specific gene deletions, insertions, or replacements in probiotic strains with greater accuracy and minimal off-target effects. This is an advancement over traditional methods but may result in unwanted mutations at other sites in the genome. Unlike traditional restriction enzymes, which cut at multiple non-specific locations, CRISPR-Cas9 can cut precisely at a designated sequence guided by RNA. This combination of gRNAs and protospacer adjacent motif sequences ensures highly specific binding and subsequent gene editing. Advanced CRISPR variants (nCas9 nickase or dCas9) can edit or regulate genes without inducing double-strand breaks, thereby reducing unwanted mutations. The CRISPR system can also simultaneously target multiple genes within a single cell by introducing multiple guide RNAs. This allows the engineering of complex metabolic pathways, such as increasing the production of multiple beneficial metabolites. From a mechanistic perspective, modifying the target location simply requires changing the gRNA sequence, which avoids labor-intensive engineering. Recent advancements have allowed the use of endogenous CRISPR-Cas systems present in the probiotic bacteria themselves, avoiding the cytotoxicity often caused by importing foreign proteins. Unlike other tools, CRISPR can be used for *in situ* applications, such as using modified bacteriophages to deliver CRISPR components directly to bacteria within the gut microbiome, allowing for the targeted elimination of pathogenic strains without killing beneficial ones [[159], [160], [161]].

## 8 Consumer perspective about GMOs and CRISPR-based probiotics

The proposed benefits of GMOs have been widely identified in laboratories on a small scale, which require further investigation to explore their potential at an industrial and commercial scale. In addition to technical and regulatory challenges, consumer acceptance remains a significant barrier to the commercialization of CRISPR-engineered probiotics, largely because of concerns regarding safety and potential environmental impacts [142]. It is also important to note that consumer resistance sometimes outpaces scientific evidence, potentially slowing the adoption of otherwise safe and beneficial innovations. Consumer acceptance of GMOs varies depending on the applications or benefits associated with genome editing. For example, higher consumer acceptance has been observed for cisgenic GM potatoes with blight resistance [142]. Moreover, consumers prefer GMOs that have enhanced health benefits over those in which the product quality has changed [143]. Bearth et al. (2024) documented consumer perceptions and acceptance of genome editing in agriculture in the USA (where GMOs have been used since the mid-1990s) and Switzerland (where GMOs have been banned since 2005) and concluded that most participants welcomed genetic innovation in agriculture. However, several contributing factors, such as lack of knowledge, complexity, and demographic location, can affect consumer behavior [142].

Currently, the rate of GMO-related product development and commercialization is low, and their expansion depends on consumer perception and acceptance [143]. People express their opinions of GMOs in various ways. For instance, some described their perceptions in terms of associated benefits and risks; the alteration of the target gene; tampering with nature; favoring naturalism, moral intuition, and acceptability; as well as their impacts on social behavior, religious aspects, and other sociodemographic properties [[144], [145], [146]]. However, the rapid increase in the world population demands food that has more properties, such as short growing time, requires fewer accessories, and has more nutritional value. To combat this, several genetic tools, including the CRISPR-Cas system, have been employed in applications in the agriculture and food industry [147]. The historical development of GMOs was summarized by Wunderlich and Gatto (2015), who documented that the USA is the leading producer of genetically bioengineered produce, accounting for 40% of global GM crops [148].

**Fig. 7:**
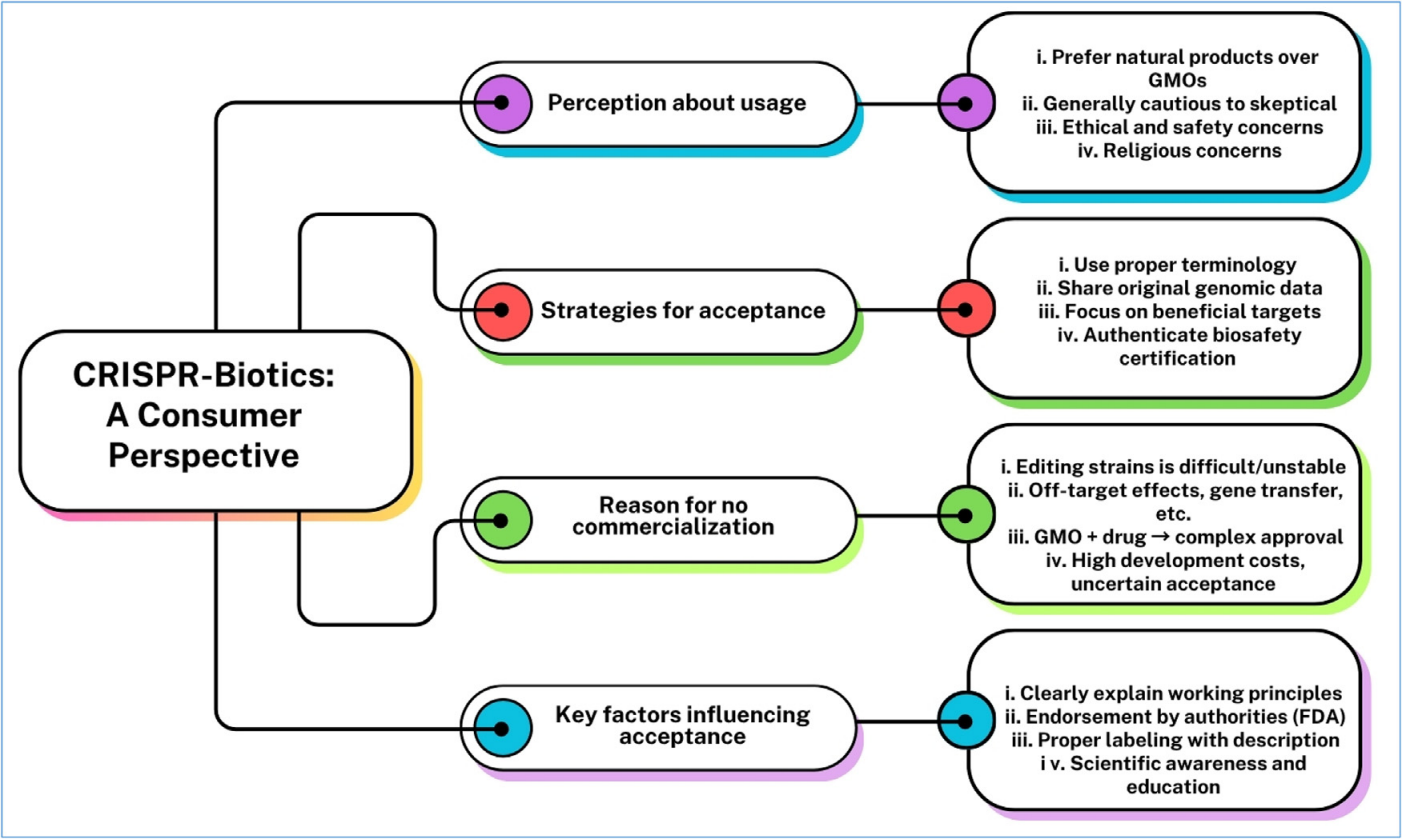
CRISPR-biotics from the consumer’s perspective.

To the best of our knowledge, no CRISPR-edited probiotic products are commercially available owing to significant obstacles related to research, safety, regulations, and ethics. However, some CRISPR-based probiotic products are already under investigation. Thus, in summary, consumers’ current perception and acceptance of CRISPR-based probiotics are cautious, and they prefer natural substances to GM probiotics. A critical view of property development *versus* the insertion of new properties or the deletion of existing properties is considered during consumers’ perceptions. The general perspective of consumers regarding CRISPR-based probiotics is summarized in Fig. 7.

For example, BiomElix® One, formulated by FOLIUM Science, uses a CRISPR-based technology platform called Guided Biotics®. This product was approved by Brazil’s Ministry of Agriculture for use as an animal feed additive and is included in the Positive List of Feed Ingredients (https://foliumscience.com/). Similarly, GeneGuard produces living biotherapeutic products, which are probiotic microbes engineered with the CRISPR-Cas system that can enhance features such as the production of bioactive molecules, biocontainment, and disease targeting. However, GeneGuard is still in the developmental stage (https://www.geneguardprobiotics.com/).

## 9 Future perspectives

The future of CRISPR-based probiotics is highly promising, with wide applications in different areas, including the food, agriculture, and medical sectors, allowing the development of novel therapeutic approaches. CRISPR-based engineering is reshaping the development and application of probiotic strains, particularly in therapeutics and personalized medicine [16,52,70]. The precision of this system allows the introduction, removal, or modification of functional traits, supporting applications ranging from metabolic regulation and mucosal adhesion to vaccine development and immune modulation [16,20]. However, the effectiveness of this tool is often strain-dependent, and its editing efficiency can differ widely among species. Future work is needed to address these variabilities by standardizing editing platforms, improving delivery systems, and expanding the range of editable hosts [14,70].

Progress is expected through multiplex editing strategies, improved plasmid design, and integration with synthetic biology and omics-based approaches [52,70]. These advancements enable scalable editing across multiple strains while minimizing off-target effects. Combining CRISPR-Cas systems with complementary genome-editing technologies may further improve precision and adaptability, particularly for complex traits related to survivability, colonization, and metabolite output [16,70]. Probiogenomics is emerging as a valuable approach for identifying and characterizing the beneficial genetic traits of candidate strains. When paired with CRISPR-based tools, this approach may enable the rational design of customized probiotics with clearly defined functions [20,106].

Another promising approach involves engineering phage-resistant strains to improve their persistence and applicability in industrial and clinical settings [106]. An important frontier is the development of next-generation probiotics, such as *Akkermansia, Faecalibacterium*, and *Bacteroides*, which have documented therapeutic potential but are difficult to culture and manipulate [149]. CRISPR-Cas systems can address these challenges by enabling targeted modifications to improve their oxygen tolerance, nutrient utilization, growth stability, and safety profiles. Tailored editing could also eliminate virulence-associated elements or introduce immunomodulatory and biosynthetic functions relevant to host health [20,70]. With advances in regulatory guidance, safety data, and host-interaction studies, CRISPR-engineered next-generation probiotics may transition from mere research concepts to viable live biotherapeutics. Continued collaboration across the microbiology, genome engineering, clinical science, and regulatory fields will determine how rapidly these developments translate into therapeutic, nutritional, and industrial applications [70,115].

There is a new horizon for the integration of CRISPR-based probiotics with artificial intelligence (AI) and machine learning (ML) approaches. AI and ML are increasingly being adopted across diverse sectors owing to their capabilities in pattern recognition, predictive analytics, automation, and data-driven decision-making. Their integration has accelerated advancements in fields ranging from healthcare and biotechnology to food systems and industrial processes, enabling optimization, precision, and scalability. However, current limitations of the CRISPR-Cas system, such as off-target effects, remain hurdles to its full utilization. The AI approach can be used to enhance gRNA design and improve gene editing efficiency. Likewise, AI can help refine nuclease-based editing, base editing, and prime editing, and thus advance the applications of the CRISPR-Cas system [155]. A similar approach can also be employed in CRISPR-based probiotics, which enhances their editing potential, lowers their off-target effects, and improves their targeted action, thus contributing to the development of next-generation probiotics with enhanced qualities [156,157].

## 10 Conclusion

The rapid advancement of genetic engineering tools and recent progress made in the field of bioengineering, particularly the CRISPR-Cas system, have allowed significant integration into probiotic research. This integration has enabled a shift from passive strain selection to targeted functional engineering, facilitating the development of strains with enhanced functionalities, including higher survivability under the harsh conditions of the gastrointestinal environment, increased mucosal adherence, and specific traits tailored for particular applications that go beyond the conventional benefits associated with probiotics. Nevertheless, the potential of CRISPR-based probiotics remains limited owing to technical challenges, strain-dependent variability, concerns regarding biosafety, and lack of clear regulatory guidelines. To overcome these barriers, future research must prioritize the optimization of host-specific editing platforms, the development of safe and effective delivery mechanisms, and implementation of rigorous ethical and regulatory standards. Progress in this field depends on strong interdisciplinary collaborations across the fields of microbiology, genomics, synthetic biology, regulatory science, and clinical research. Public perception is an equally important determinant of the success of this technology. Despite the technological promise of CRISPR-based probiotics, their acceptance remains uncertain owing to longstanding concerns surrounding GMOs. Misconceptions, limited awareness, and the belief that CRISPR-edited strains are “unnatural” contribute to skepticism. Moreover, concerns related to safety, ethics, and unintended ecological consequences can further hinder clinical translation and market adoption, even when scientific data support efficacy and safety. Addressing these concerns through transparent communication, evidence-based regulations, and public engagement is essential to ensure that CRISPR-based probiotics advance from laboratory innovations to real-world applications.

## CRediT authorship contribution statement

**Abrar Hussain:** Writing – review & editing, Writing – original draft, Investigation, Formal analysis, Data curation, Conceptualization. **Naheed Mojgani:** Validation, Methodology, Formal analysis, Data curation. **Maria Fareed Siddiqui:** Writing – review & editing, Visualization, Software, Methodology. **Mohammad Inam Khan:** Writing – review & editing, Validation, Methodology, Data curation. **Syed Abid Ali:** Writing – review & editing, Validation, Supervision, Conceptualization.

## Declaration of competing interest

The authors declare that they have no known competing interests that could have appeared to influence the work reported in this paper.
